# Harvard Odontological Society

**Published:** 1896-04

**Authors:** 


AMERICAN JOURNAL

—OF—

DENTAL SCIENCE.

VOL. XXIX. BALTIMORE, APRIL, 1896. NO. 12.

ARTICLE I.

### HARVARD ODONTOLOGICAL SOCIETY.

The regular monthly meeting of this society was held March 28, 1895, at Young's Hotel, President Dr. James Shepherd in the chair.

After the usual business meeting, Dr. Julius G. W. Werner read a paper upon the "Treatment of Decay in Approximate Surfaces of Bicuspids and Molars."

The following is an abstract of the discussion :

Dr. Stanley : I would like to ask Dr. Werner if his principal objection to the use of amalgam is the effect of discolored teeth ?

Dr. Werner : My principal objections are the discoloration to tooth-substance, the homœopathic objection, the want of edge-strength, and arresting decay no better than gold.

Dr. Stanley : Does Dr. Werner contend that gold has a more preservative effect on the dentine of a tooth than amalgam ?

Dr. Werner : I think it has fully as much, is nicer, cleaner, is a pure metal, hence more desirable than an alloy or a mixture of silver, tin, and mercury.

Dr. Stanley: Then why do you recommend tin in some instances?

Dr. Werner: Because we get an oxidation of the tin, a benefit, with no discoloration of tooth-substance, nor presence of mercury, as we have in amalgam.

Dr. Stanley: The amalgam that I use has a good percentage of tin in it. As I understand it, gold exerts only a mechanical effect on a tooth. In a combination filling, with amalgam at the cervical wall, the result will be better than from amalgam alone.

Dr. Werner: What makes you think that?

Dr. Stanley: The gold will remove all excess of mercury, and give it a better edge-strength.

Dr. Werner: Do your patients prefer amalgam?

Dr. Stanley: They prefer what I advocate, I do not advocate any one filling material over another, but the best material must be used for the case, according to my judgment, and that is what my patients accept.

Dr. Werner: Don't you think your judgment is often dictated by the amount of time, money, skill and labor connected with a case where you use amalgam?

Dr. Stanley: I look to the result to be obtained first, and then use what I think is going to serve my purpose best. It isn't necessary to fill the cavity four-fifths with amalgam. It is difficult to pack gold under the gum, and in a great many cases a plastic filling gives much more satisfactory results. These contour fillings for such cases are ideal on paper, but in a few years I think they will fail quicker than a combination with amalgam instead of gold alone.

Dr. Werner: Did you ever fill to contour a bicuspid with gold.

Dr. Stanley: You must be Joking, doctor; certainly I have. I have been in practice for ten years.

Dr. Werner: And has it kept it from decay?

Dr. Stanley: Yes.

Dr. Werner: Are you not satisfied with that?

Dr. Stanley: Yes; but I claim I have progressed from that. From all gold I have come to use combination fillings, which, in their proper places, are superior and more easily and expeditiously adapted.

I have used a steel matrix for a long time in the filling of approximal surfaces in bicuspids and molars, using soft gold at the cervical wall—sometimes absolutely non-cohesive gold. I use mainly, however, soft gold as it comes to us in cylinders; it can be made slightly cohesive by annealing. I have in a few cases used tin and gold at the cervical wall, but I do not recognize great advantage in tin and gold, excepting in very poor teeth.

There are cases where I would use amalgam at the cervical wall, though I have never practiced that method much. By the aid of the matrix I find it a comparatively simple matter to fill cavities of this kind, using a large percentage of soft gold about the walls and on the approximal surface, and what more satisfactory material is there at present in use? Who of us that have been in practice twenty years have not taken out non-cohesive gold fillings which have been in many years, and, although they were very ill-looking fillings, have been surprised to find how nicely the dentine has been preserved. The edges of the cavity have been rough and uneven, and the filling unsightly, and we have been prompted to take it out, perhaps, because of its unsightliness, but we have found that soft gold has preserved the tooth, and in strong teeth I do not know what better material we can use.

One who is not familiar with this method may perhaps imagine that it is a very tedious or laborious operation to fill a good sized approximal cavity in a bicuspid or molar. It *is* more trying than with a plastic, but the result is so much superior, so much more comfortable to the patient, that it would seem to me to very much outweigh the time and labor saved by putting in a plastic, which must of necessity be temporary. Really, the greater part of that filling can be placed in without the mallet and in rather large pieces after you commence, and I am surprised when I compare the time that it takes me now to place in a large compound bicuspid or molar filling to what it did when I first commenced. If I could have had the knowledge regarding this method at that time that I have to day, it would have saved me a great many hours of hard work and my patients much pain.

In regard to the matter of platinum and gold on the surface, I rarely use it, excepting where I am building down teeth that come to much wear. In cases where the molar teeth are gone, and the front teeth have to do double service, all the mastication being done by the front teeth, the gold will wear very rapidly. In those cases platinum and gold for the surface is very much more durable than pure gold. I have used platinum-gold in approximate surfaces in front teeth, but do not now. It is a little better color, perhaps, but where you get two approximal cavities coming together, the reflection is such as to give a dark appearance,—more than the pure gold,—so that my use of platinum and gold is very limited. I do not see any objection to using tin and gold if it is where it does not show even in the very strongest teeth.

Dr. Werner: There is one paragraph I wish particularly to call Dr. Briggs's attention to—namely, "where good judgment and favorable conditions dictate a permanent in distinction from a temporary or plastic filling." That covers, I think, all the questions of judgment that can be brought up. I am not to suppose the case in question and good judgment as both being absent. I have no doubt you use good judgment in your practice and you must not get the idea that I do not in mine. I believe firmly in the matrix, full contour, with gold and tin at the cervix, and not in amalgam.

Dr. Stanley: Do you use amalgam at all in your practice?

Dr. Werner: Very little.

Dr. Stanley: Supposing we take the posterior surface of a second molar, with the wisdom-tooth in position; it is decayed well up under the gum, and the tooth will stand a metal filling—how do you fill it?

Dr. Werner: I would fill such posterior surface in a second molar with gold, using at the cervical wall gold and tin, restore it with the use of the matrix to full contour, do it in a comparatively short time with comparative comfort to the patient.

Dr. Stanley: Of course, when a man has a theory, or hobby, or pet method, he becomes in a greater or less degree master of the technique that it requires to execute it, and I can understand how Dr. Werner, from his constant practice, might put in such a large contour filling, and build it up from the cervical wall with gold, in less time perhaps than the operator who does not believe in filling such cavities with gold alone. But I certainly could do it a great deal easier for myself and my patient, and I should have more confidence in the service of the filling, if it had amalgam at the cervical wall.

Dr. Werner: I think I am doing a better service for my patients when I restore an approximate surface to full contour with gold and tin at the cervix, than if I used amalgam, gutta-percha or cement, both as to arresting decay and general desirability.

Dr. Stanley: I should not for a moment think of putting cement in such a place, but amalgam for that place would be a permanent filling.

I believe most thoroughly in the use of the matrix, and I use it sometimes, even when I am putting in gutta-percha or cement fillings, for the preparation of approximal cavities in bicuspid teeth where I do not think they are ready for a gold filling. The success of those fillings, I believe, largely depends on the preparation of the cavity. The cavity should be just as thoroughly prepared for one material as another. The standard alloy hardens very quickly, and by the time you have finished the filling with gold, the amalgam has become so hard that you can polish with a strip without affecting the gold, and you have your contour fillings just the same.

Dr. Werner: How much time do you spend on a surface of that kind to fill with amalgam?

Dr. Stanley: I could not say—twenty minutes to half an hour, or longer; it depends on the case,

Dr. Werner: How much time afterwards in finishing?

Dr. Stanley: That also depends on the case. I finish it to the contour of the tooth. The matrix holds it there until it becomes hardened.

Dr. Werner: What becomes of the contour after you you remove the matrix?

Dr. Stanley: It stays there.

Dr. Werner: You can't finish the amalgam filling at that sitting?

Dr. Stanley: Oh, yes, you can; a thin, narrow strip will do it.

President Shepherd: I believe there is in the room a gentleman who has in his mouth an example of some of the work we have heard about to night. Will the gentleman tell us something about it?

Dr. Cheney: I don't know that I can say anything further than that they have been perfectly satisfactory and comfortable. I should be glad to show them to any one who cares to see them.

Dr. Ainsworth: I might explain that those fillings were put in at a clinic before the Vermont Society under this method five or six years ago, and I did not feel very sanguine of the results. One of them especially was very difficult, one I do not think a person has a license to clinic with and expect it to speak well for the method. It was in a superior bicuspid, well under the gum, with the nerve exposed, and I was in doubt at that time whether we should have trouble. The conditions were such that it was impossible to get the rubber on without putting on the matrix first. I should like to hear if that tooth has given any trouble since.

Dr. Cheney: I am glad to state that the root has never given me any trouble since the filling was put in. If you will remember, the pulp was first covered with varnish and cement.

Dr. Ainsworth: I remember there was something placed in to protect the pulp. It was thoroughly exposed, and I felt it was treading on delicate ground to fill such a tooth without removing the pulp.

Dr. Cheney: I have been very much interested in the paper of Dr. Werner, and am a thorough believer in contour work to protect the gingival margin. I am also quite a believer in the combination work. I believe a great many times; by using amalgam at the cervical wall, a very difficult case can be handled easier than with gold entire. In placing in those approximal fillings I have never used a matrix, but I have used soft gold at the cervical wall, and burnished it over the edge as well as I could, and then come down with slightly cohesive gold. I have seen some sore gums from not having the filling properly contoured —in fact, I had one or two cases in my own mouth which gave me considerable trouble from food working up there.

Dr. Chandler: Three or four years ago I was of the opinion that in a great many cases an amalgam filling was as good as a gold filling—I mean as regards restoring contour and comfort to the patient; but since I have seen Dr. Werner's work, I am very strongly in favor of the use of gold. It seems strange to me now that an operator should think of placing amalgam into those approximal cavities, leaving the edges of the cavities perfectly straight up and down, affording an opportunity for food to collect, and the edges of the cavity to become broken down to expose the dentine, when a full contour gold filling will not only protect the edges of the cavity and prevent the progress of decay, but be a comfort to the patient.

President Shepherd: Will Dr. Werner tell us why he prefers hand-pressure to the pressure of the mallet in connection with soft gold?

Dr. Werner: Because you condense the soft gold in a manner more comfortable to the patient. Hand-pressure is more thoroughly adapted to the condensation of the gold than the quick, hammering blow of the mallet, and with the assistance of the matrix the result is everything that could be desired.

President Shepherd: Is it as solid as a hammered gold filling?

Dr. Werner: I don't think that the hammered solidity is an advantage to the tooth; I think it is a decided disadvantage. I would, of course, hammer the very last portion of the gold, the portion that comes to the occluding surface, but nearly eight-tenths of the filling I would do with hand-pressure. When you learn how to use the matrix skillfully, the cervical wall portion that many now fill with amalgam they will fill with gold and like it better. I am not a believer in the Hanhemannian theory, but I do not like a filling of which mercury is an ingredient. There is a member here to-night who a year ago spoke of a case where he removed thirty-five amalgam fillings and replaced them with gutta-percha and cement. I have never in all my experience, which, including pupilage, covers nearly twenty-three years, seen a case where good judgment would seem to dictate the insertion of thirty-five gutta-percha fillings in one mouth. My professional sense of duty dictates to me, if the patient is willing and the tooth is strong enough, to put in the best and make the most lasting filling I can. Look at this typical case here, where the patient became absolutely disgusted with the imbecility of her previous dentist, who filled that approximate bicuspid surface with cement every six months, or year and a half at the longest. It was never thoroughly excavated. I think she has paid more for those oft-repeated cement fillings than a thorough full-contour matrix gold filling would have cost, and she has had none of the comfort and the protection of the gum that she would have had with a contour gold filling.

Dr. Stanley: In what form do you use your gold?

Dr. Werner: I use Williams's soft burnished gold cylinders. It is very pliable and gives a beautiful finish. It can be thoroughly condensed, and to me it is the most satisfactory for use with the matrix. I do not like a gold that is absolutely non-cohesive.

President shepherd: Combination fillings have been referred to several times this evening. Perhaps Dr. Clapp will say a few words to us on that subject.

Dr. Clapp: I don't think it is necessary for me to say anything about combination fillings. You know how heartily I believe in them, and, as I have often said, it seems to me that I could not practice without them.

In regard to the paper, I have nothing to say except to offer my thanks for it, especially for that part where Dr. Werner insists on separating the teeth properly, and getting at them so that contour fillings can be thoroughly and perfectly adapted to the cavity. In my opinion, that is the cause of the failure of the majority of such fillings, in not giving time for preparation. You cannot make a proper filling in an improperly prepared place, and I freely admit that my failures come oftener from the lack of time to separate the teeth so that the proper contour fillings may be placed in the cavities than from any other cause. It seems strange that to-day anything but a contour filling in approximal cavities should be thought of or be tolerated, and conversely, I believe we are gaining in our knowledge of what should be done, and in our desire to do it, and in our skill to do it; and also I believe we are gaining in the appreciation of our work by our patients, and that is very gratifying. There is no limit to be placed upon the value of teeth to the person who owns them, and it is a fault that that we should all strive to overcome, this hurrying matters too much.

I heartily endorse the paper, so far as its advocacy of contour work is concerned. So far as selecting the material enters into the case, each operator must decide for himself upon the circumstances presented. I can do better work in some instances with amalgam combined with gold than with gold alone. I do not like to use it, but I am compelled to because it is the best thing that I have in many instances for the case at hand.

President Shepherd: If no other gentleman cares to speak on this subject, I will ask Dr. Werner if he wishes to say anything in closing.

Dr. Werner: I have not said half that I could say on this subject, but may have said so much as to have tired some here present. I spoke but little in my short paper on the preparation of cavities,—almost nothing,—but that is quite an item, and I am glad that Dr. Clapp sees the vital point. He has the truth of the matter—your contour and your preparation of cavity is the secret of success, and that can only be secured with the matrix.

Dr. Stanley: It seems to me that the preparation of the cavity is independent of the matrix.

Dr. Werner: In all of the cases that I have presented to-night I could not have excavated and filled with gold without the use of the matrix. The excavation and preparation of the cavity many times takes one a good half of the whole length of time of the operation. I have no patience with the unprofessional plastering and hurrying method that is sometimes practiced.

If I should tell you that since the first day of January until to-day—and I have operated every day in the week except Saturday afternoons and Sundays—I have used amalgam perhaps six or eight times, you would be surprised; you would hardly believe it. Another operator will use it five or six times in one sitting, and do that same thing six or eight times in his daily work. We must make allowance for different practices, to be sure, but also for an immense difference in the willingness, the skill and the ability of operators to perform certain kinds of operations. *International Dental Journal.*

